# A case report: Clinical application of celiac plexus block in bile duct interventional procedures

**DOI:** 10.1097/MD.0000000000004106

**Published:** 2016-07-08

**Authors:** Myong-Hwan Karm, Hyun-Seok Cho, Jae-Young Lee, Heon-Yong Bae, Ho-Soo Ahn, Yeon Ju Kim, Jeong-Gil Leem, Seong-Soo Choi

**Affiliations:** Department of Anesthesiology and Pain Medicine, Asan Medical Center, University of Ulsan College of Medicine, Seoul, Republic of Korea.

**Keywords:** biliary tract, celiac plexus block, dilatation, drainage, intervention, visceral pain

## Abstract

Although percutaneous transhepatic biliary drainage (PTBD) and tract dilatation (TD) are very painful procedures, almost all of those procedures have been conducted under local anesthesia and opioid injection due to the lack of manpower and time. Celiac plexus block (CPB) is an interventional technique used for diagnostic and therapeutic purposes in the treatment of abdominovisceral pain. CPB decreases the side effects of opioid medications and enhances analgesia from medications. We present the case of a patient who underwent PTBD and TD under CPB in order to reduce procedure-related abdominal pain.

CPB can be a useful alternative technique for pain management during and after biliary interventional procedures, although CPB-induced complications must always be kept in mind.

## 1 Introduction

Bile duct stones cause inflammation of the biliary system and jaundice.^[1]^ Most patients with acute calculous cholecystitis have had attacks of severe pain often referred to as biliary colic^[2]^ and which is caused by the obstruction of the gallbladder neck by a bile-duct stone.^[3]^ When conservative treatment, rather than cholecystectomy, is selected, the patient is treated with antibiotics and intravenous fluids during the acute phase. The patient then undergoes procedure for symptom improvement and stone removal, such as percutaneous transhepatic biliary drainage (PTBD)^[4]^ with or without tract dilatation (TD). Pain management during these bile-duct procedures is always challenging. Although PTBD and TD are very painful procedures, they are commonly conducted at our hospital with the patient under moderate sedation using local anesthesia and opioid administration. An ultrashort-acting opioid can also be utilized for the management of painful radiologic procedures.^[5]^ However, it is well-known that opioid administration can lead to side effects such as pruritus, delirium, sedation, respiratory depression, altered gut motility, sphincter of Oddi spasm, and constipation.^[6]^ Therefore, these opioid side effects may limit the sufficient administration of opioid for pain control.

Celiac plexus block (CPB) is an interventional procedure used for diagnostic and therapeutic purposes in the treatment of visceral pain in the upper abdomen.^[7]^ The main indication for CPB includes visceral abdominal pain refractory to analgesic interventions, and which emanates from the pancreas, stomach, liver, biliary tract, kidney, small intestine, colon, and adrenal glands.^[8,9]^ These types of pain have been treated with CPB in order to decrease theoretically the side effects of opioid medications and to enhance analgesia caused by medications.^[7]^ Here, we present the case of a patient who underwent PTBD and TD under CPB in order to reduce biliary procedure-related abdominal pain.

## 2 Case report

**Figure 1:**
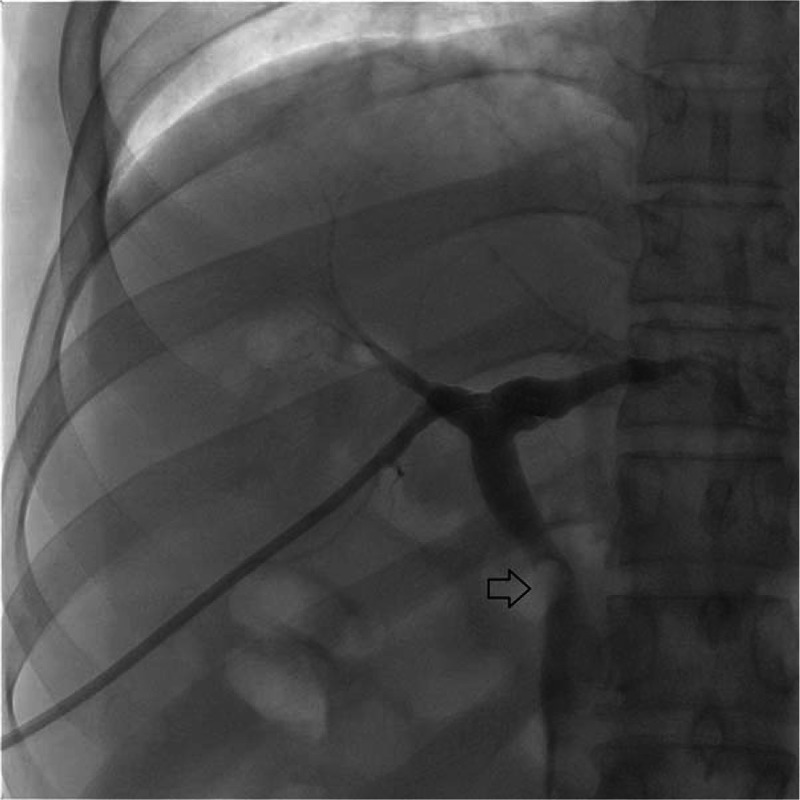
Fluoroscopy image after initial percutaneous transhepatic biliary drainage (PTBD). The stone (arrow) in the proximal common bile duct still remained after PTBD.

A 37-year-old male patient (167 cm; 74 kg, no past medical history) was admitted with symptoms of fever (37.7 °C), RUQ pain, jaundice, nausea, and dyspepsia. The laboratory examinations revealed the following: serum total bilirubin 7.5 mg/dL; direct bilirubin 5.0 mg/dL; AST 168 IU/L; and ALT 257 IU/L. The computed tomography findings showed a 1-cm, dark signal intensity lesion in the proximal common bile duct (CBD) as well as bile-duct obstruction. The patient was diagnosed with acute cholecystitis with a CBD stone and was planned to be performed endoscopic retrograde cholangiopancreatography. The stone removal with endoscopic retrograde cholangiopancreatography failed due to a distal CBD stricture and the patient underwent emergency PTBD for symptom improvement. After the PTBD, as the patient complained of severe abdominal and PTBD insertion site pain (numerical rating scale [NRS] 8–9), pethidine (25 mg) was injected 3 times. Following the pethidine injections, as the patient complained of severe nausea with vomiting, metoclopramide (10 mg) was injected. However, the stone was not removed after PTBD due to a CBD stricture (Fig. 1). The interventional radiologist planned TD with a PTBD procedure and consulted our Department in order to manage the patient's pain during the intervention.

**Figure 2:**
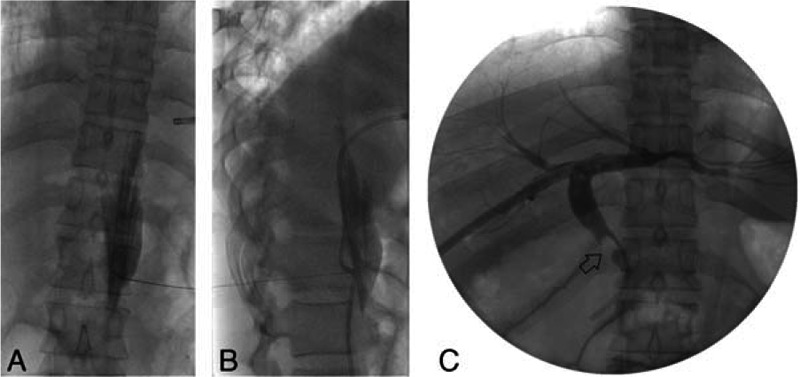
Anteroposterior (A) and lateral (B) fluoroscopy image obtained during the celiac plexus block (CPB) during tract dilatation (C). In the prone position, the right side of the CPB was performed using a posterior retrocrural approach utilizing a bent tip needle with 0.25% bupivacaine (10cc) under fluoroscopy (A and B). The patient was changed into the supine position and underwent tract dilatation and percutaneous transhepatic biliary drainage. The common bile duct stone (arrow) is shown as a filling defect in the posteroanterior view (C).

As the patient was reluctant to receive further opioid administration and did not want to experience nausea and vomiting after the opioid administration, we initially planned a thoracic epidural block after epidural catheter insertion without opioid administration. However, the patient refused during the process of obtaining informed consent due to the possible side effects of a thoracic epidural block. Therefore, we considered CPB for his right upper abdominal pain during the TD and PTBD procedure. After the patient was informed regarding and understood the effects and possible complications of both thoracic epidural block and CPB, he decided to undergo CPB regardless of the possibility of complications. Skin infiltration for local anesthesia was performed using 1% lidocaine and with the patient in the prone position. Right-side CPB using a posterior retrocrural approach at the T12-L1 level and using a bent tip needle^[10]^ with 0.25% bupivacaine (10cc) under fluoroscopy was performed in order to reduce the patient's pain during and after TD and the PTBD procedure (Fig. 2A and B). The patient was changed into the supine position and underwent TD and PTBD without severe pain or inconvenience (Fig. 2C). Although TD was in progress, ephedrine (5 mg) was injected once and crystalloid (350 mL) was infused due to hypotension (70/40) which immediately recovered. Other postprocedural complications were not observed. He was well tolerable and did not complain of severe pain during procedures.

After transfer to the ward, the patient also did not complain of right upper abdominal pain. But the patient complained of moderate pain (NRS 5) at the PTBD insertion site, tramadol (50 mg) was injected only once. Mild insertion site pain (NRS 3) continued for approximately 1 day after TD and the PTBD procedure.

Although the patient will be planned to discharge after TD and PTBD procedure, discharge was postponed due to fever of the patient. After 14 days of antibiotic treatment, the stone was removed using percutaneous transhepatic choledochoscopy. The patient was discharged 1 week later without any complications or pain.

## 3 Discussion

Patients with acute cholecystitis with CBD stone suffer from inflammation, jaundice, and biliary colic.^[1,2]^ The procedures (PTBD and TD) for symptom improvement and CBD stone removal are the cause of the pain.^[4]^ As opioids are commonly used for pain control during these procedures,^[5]^ we must consider the side effects of the opioids. Our patient was already suffering from PTBD procedure pain and a radiologist consulted to our department regarding his intractable abdominal pain management. With our institutional protocol for this situation, monitored anesthesia care is often performed with an opioid in addition to propofol or dexmedetomidine. However, the present patient did not want opioid treatment and sedation during the procedure because he experienced severe nausea and vomiting after opioid administration. Although we considered thoracic epidural block after epidural catheter insertion for pain control during the biliary procedure and after the procedure, the patient refused the thoracic epidural block. Therefore, we considered right side CPB for pain control during the biliary procedure. Indeed, CPB is a valuable adjunctive therapy for managing upper abdominal pain associated with chronic visceral pathology.^[7]^ Right side CPB using a posterior retrocrural approach has successfully reduced pain during PTBD and TD, and only transient hypotension was observed during the procedure. Furthermore, post-TD pain was also controlled without additional opioid administration even with a remaining stone. This is our first experience indicating that CPB may be a useful alternative method for pain control during a bile duct interventional procedure.

The present case report is of significance in that CPB during a bile duct interventional procedure can reduce pain and opioid overdose without severe complications. CPB can be a useful alternative technique for pain management during and after a bile duct interventional procedure, although CPB-induced complications must always be kept in mind.

## 4 Informed consent

This study adhered to the tenets of the Declaration of Helsinki. The patient signed informed consent for the publication of this case report and any associated images.
